# Are multiple limb injuries in severely injured patients negligible? Evaluation of progression and outcome using a new scoring system for extremity injury: the Extremity Severity Score (ESS)

**DOI:** 10.1007/s00068-025-02987-4

**Published:** 2025-10-28

**Authors:** Claudius Thiedemann, Markus Rupp, Anton Heller, Julia Lenz, Ingmar Geiger, Daniel Popp, Volker Alt, Antonio Ernstberger

**Affiliations:** 1 Clinic and Polyclinic for Trauma Surgery, University Hospital Regensburg, Franz-Josef-Strauss-Allee 11, 93053 Regensburg, Germany; 2 Clinic and Polyclinic for Cardiac, Thoracic and Cardiovascular Surgery, University Hospital Regensburg, Regensburg, Germany; 3 Professor of Marketing and Statistics, School of Management & Health, Aalen University, Aalen, Germany

**Keywords:** Multiple trauma, Severely injured patient, Limb injury, Extremity Severity Score, Polytrauma, Major Trauma

## Abstract

**Introduction:**

Extremity injuries appear to have less impact on the mortality of multiple trauma patients (ISS ≥ 16). The Primary Survey of the Advanced Trauma Life Support (ATLS) only lists pelvic and femur fractures among the extremity injuries. The aim of this study was to evaluate the role of multiple extremity injuries in terms of lethality, progression, and complications in multiple trauma patients and the actual influence of concomitant blood loss. The Extremity Severity Score (ESS) was developed as a central instrument for this purpose.

**Material & methods:**

This investigation is a retrospective single center study at a Level I trauma center over the period 2008–2019. The study cohort was identified as patients who met an Injury Severity Score (ISS) of at least 16. People who were < 16 years old at the time of the accident, patients who were primarily treated in other hospitals or transferred directly from the trauma bay, and cases without a Revised Injury Severity Classification Score 2 (RISC2) were excluded. Similar to the calculation of the ISS or NISS, the three most severe limb injuries (including the bony pelvis, corresponding to the ISS region of the extremities) were squared and added together to calculate the ESS. The study cohort was divided into the groups ESS ≥ 16 and ESS < 16 and these were examined with regard to the primary endpoint of lethality and several secondary endpoints. In addition to the univariate analysis of the data set, a logistic regression model was calculated.

**Results:**

Out of 3.101 cases 1.227 patients and 5.824 extremity injuries met the inclusion criteria. Both unadjusted lethality and Standardized Mortality Rate (SMR) were not significantly different for the EES < 16 vs. ESS ≥ 16 group overall (22.5% vs. 18.0%; 0.97 vs. 0.84, *p* > 0.05). Patients in both groups died most frequently from Traumatic Brain Injury (TBI) (72.9%/47.4%), followed by exsanguination (9.8%/19.3%) and Multi Organ Failure (MOF) (6.8%/17.5%). More patients in the ESS ≥ 16 group died of exsanguination (4.6% vs. 23.1%, *p* = 0.007), while patients in the ESS < 16 group died more frequently of TBI (77.0% vs. 30.8%, *p* = 0.002). For the secondary endpoints, there were significantly more surgical interventions (2.5 vs. 7.6, *p* ≤ 0.001), an increased blood transfusion rate (20.3% vs. 50.6%, *p* ≤ 0.001) and longer ICU (8.9 d vs. 12.1d, *p* ≤ 0.001) and total hospital stay (8.9 d vs. 12.1 d, *p* ≤ 0.001) for the ESS ≥ 16 group.

**Conclusion:**

In this study multiple severe extremity injuries did not influence lethality but the clinical course. ATLS is right for the first moment. However, treating more extremity injuries requires more resources. The result of comparable lethality can only be achieved, if a hospital can provide these resources for this vulnerable patient group. Patients with injuries to several extremities therefore still require special attention.

## Introduction

Due to the high number of accidents and the direct consequences for the oftentimes young patients, the care of multiple trauma patients is of great importance. The WHO considers trauma to be part of the global burden of disease and, following the “Decade of Action for Road Safety 2011–2020”, has now proclaimed an additional ”Decade of Action for Road Safety 2021–2030” “to halve deaths by 2030” [1–3]. Extremity injuries appear to play a subordinate role in terms of mortality, except for pelvic and femur fractures. Both the ATLS [4] and the German S3 guideline on the treatment of patients with severe/multiple injuries [5, 6] only focus on extremity injuries with severe blood loss, which can impair vital functions.

Until now, the priority has been to avoid further damage and not delaying the overall rescue time by treating extremity injuries, particularly in the prehospital phase [7]. However, it has been shown that heavy bleeding is the cause of death in severely injured patients in around 30% of all cases [5]. In their study, Buschmann et al. showed that unrecognized or insufficiently stopped blood loss is also the most common preventable cause of death [8–10]. As already described in the manual of the National Association of Emergency Medical Technicians, this type of bleeding typically originates from arteries in the extremities [11], bleeding sites are found along the long proximal tubular bones, of which the femur is particularly noteworthy [4, 11]. In the meantime, the recommendation to stop bleeding, which is well established in the military, has also found its way into the civilian emergency services, which underlines its importance [12, 13].

In an unstable patient, the examination of the extremities is sometimes neglected and injuries are overlooked [14, 15]. According to the study by Enderson et al., these are retrospectively found in the extremities, particularly in multiple trauma patients, and often require surgical treatment [16]. There is still no standardized clinical assessment tool for the evaluation of relevant lower extremity injuries [17]. However, there are newer studies that deal specifically with the examination of the lower extremity. Berk et al. have already demonstrated positive results using their examination method for larger fractures of the femur and tibial shaft [17]. As Ruchholtz et al. demonstrated, the duration of the trauma bay time influences the treatment results and the morbidity/mortality of a severely injured patient [18]. The extent to which multiple extremity injuries, including those beyond the thigh, lead to a reduction in the probability of survival or to an increased complication rate has not yet been investigated in detail.

A separate extremity scoring system was necessary to record multiple limb injuries.

The ISS score can, if at all, only take into account the most severe extremity injury (consideration of the three most severely injured body regions, each with the most severe injury) [19]. The NISS includes the three most severe injuries, regardless of the body regions. Given that this study was based on a population of severely injured patients, it was expected that the NISS would also include a maximum of one extremity injury [20]. Thus, the ESS was created.

The aim of this study was to investigate the impact of multiple extremity injuries with a new scoring system (ESS) in severely injured patients (ISS ≥ 16) on lethality as a primary endpoint and secondary endpoints in the clinical course. In addition, the risk and influencing factors associated with multiple limb injuries should be assessed.

## Materials and methods

Our study is a retrospective single center study at a Level I Trauma Center (German: ÜTZ) over a period of 12 years (2008–2019).

The evaluated data set included the items from the TraumaRegister DGU^®^ [21] and an in-house data set with an additional 350 variables, which described the preclinical and traumaroom phases more precisely. The data in the trauma bay were collected prospectively by study assistants. The complete data set documents the entire primary course from trauma to discharge or death. The treatment phases pre-hospital, trauma bay, intensive care unit and outcome were precisely mapped in the data set with measures, medication and infusion/transfusion volumes, time courses and vital parameters. While plausibility checks were already carried out during data entry in the TraumaRegister DGU^®^, all data records were checked and improved according to the dual control principle before release in order to achieve the best possible data quality.

Data from preclinically deceased patients could not be collected.

The Glasgow Coma Scale was used preclinically to describe the neurological deficits [22]. The injury severity of a single injury was represented by the Abbreviated Injury Scale (AIS) [23], the overall injury severity in the case of multiple injuries by the Injury Severity Score (ISS) or the New Injury Severity Score (NISS) [19, 20, 24]. The expected/calculated mortality was estimated using the Revised Injury Severity Classification Score 2 (RISC2) [25].

Cases were included if the injury severity according to the Injury Severity Score (ISS) was ≥ 16 [19, 24]. Patients were excluded if they were younger than 16 years at the time of the accident, were primarily treated in other hospitals, or were transferred directly from the trauma bay of the study hospital. Cases with missing information that was required to calculate the Revised Injury Severity Classification Score 2 (RISC2) could also not be included [25].

In order to enable adjustment and comparison of the limb injuries, a new scoring system was developed specifically for this study: Similar to the calculation of the NISS [20], the AIS values of the 3 most severe extremity injuries were squared and added together. Extremity fractures were defined as injuries occurring in the ISS region extremities, including pelvis and femur fractures.

$$ESS=\left(AIS_{limb1}\right)^2+\left(AIS_{limb2}\right)^2+\left(AIS_{limb3}\right)^2$$

Analogous to the ISS and the NISS, in cases with less than three extremity injuries, the existing one or two extremity injuries were calculated using the formula.

The Extremity Severity Score (ESS) allows for the first time to describe an extremity combination injury with a numerical value. The study population was divided into the groups ESS ≥ 16 and ESS < 16. In a second step, the two sub-samples were further subdivided according to age. Here, both groups were divided into the subgroups age ≥ 50 years and age < 50 years in order to shed more light on the different causes of trauma and injury patterns of the younger and older traumatized individuals.

The cut-off was chosen to form two groups of similar sizes. The frequency distributions of age within the groups were also similar, with two peaks among the younger accident victims in both groups.

For statistical evaluation, the chi-square test was used for binary or ordinal data and the Kruskal-Wallis test for metric variables. The variables evaluated in the sample were not normally distributed according to the Kolmogorov-Smirnov test. A *p* < 0.05 was considered statistically significant.

In addition, a logistic regression model was calculated. On the one hand, variables that had proven to be significant in previous calculations were included in this model, and on the other hand, data from the current literature revealed known risk factors for death in multiple trauma patients. TBI is a known risk factor, which was mapped using the AIS value. In addition, the ESS was ordinally scaled (ESS ≥ 16 and ESS < 16) and included in the model. The preclinical shock, defined as a systolic blood pressure ≤ 90mmHg, the GCS value and the need for mass transfusion were the clinical variables. The dependent variable was the dichotomous variable of death. Binomial logistic regression was calculated to examine the extent to which the above factors contributed to death. The statistical analyses were calculated with IBM SPSS 23. The study was approved by the Ethics Committee of the University of Regensburg (number 24–3718−104).

## Results

**Fig. 1:**
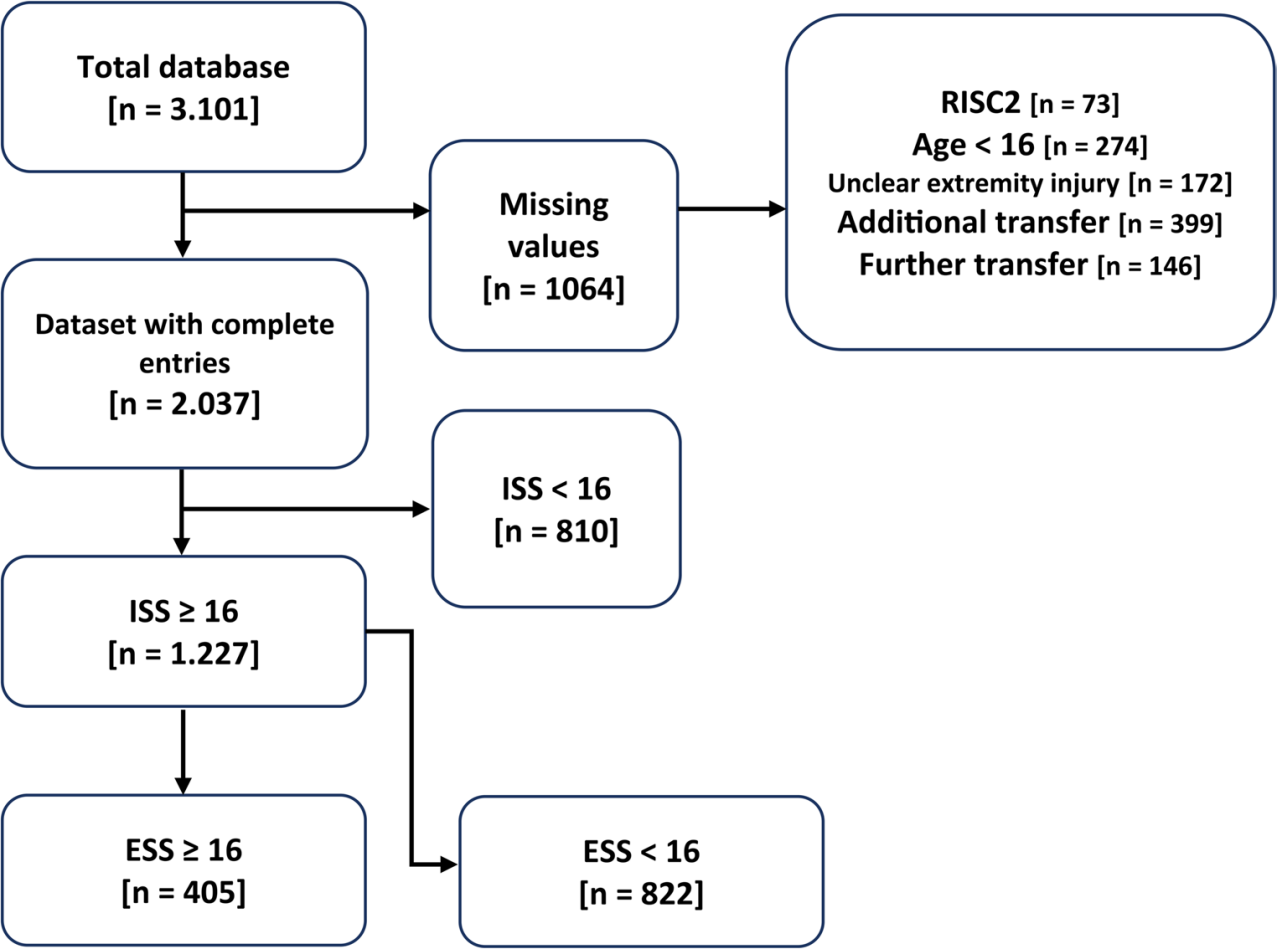
Breakdown of the study population by inclusion and exclusion criteria and their calculation, RISC2: Revised Injury Severity Classification 2, ISS: Injury Severity Score, ESS: Extremity Severity Score

**Fig. 2:**
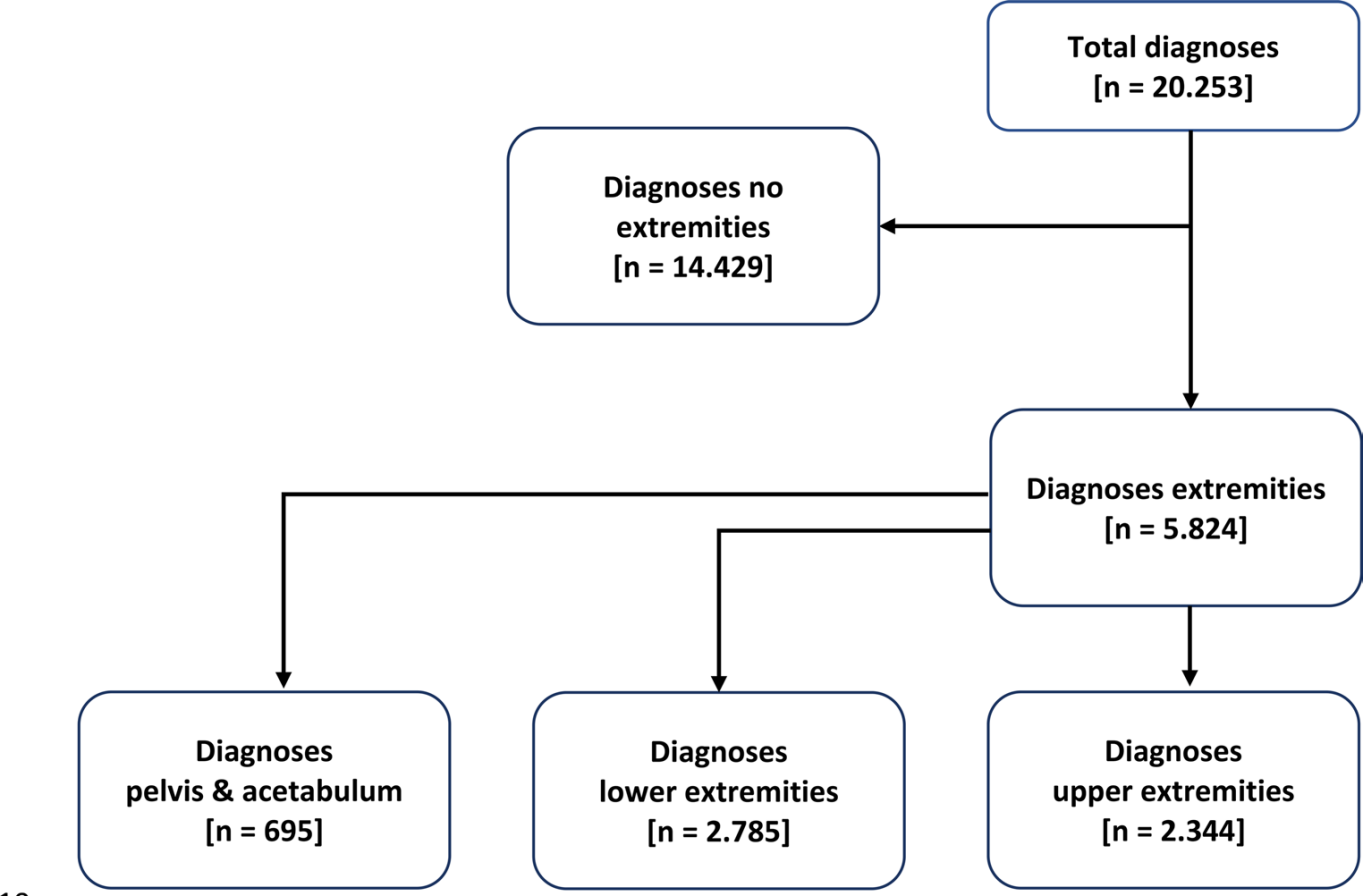
Calculation of the diagnoses of all extremity injuries and their distribution among the study population

The calculation basis for this study was a database with a total of 3.101 cases. After applying the inclusion and exclusion criteria (Fig. 1), 1.227 patients and 5.824 injuries of the extremities (Fig. 2*)* were identified.

The ESS group ≥ 16 included 70.80% men (*n* = 287) and 29.29% women (*n* = 118).

The study group ESS < 16 included 74.30% (*n* = 611) men and 25.70% (*n* = 211) women. The mean age of the ESS ≥ 16 group was significantly younger than in the ESS < 16 group (ESS < 16: 48.32 years vs. ESS ≥ 16: 42.16 years; *p* ≤ 0.001).

A further analysis of the study population based on age (< 50 years or age ≥ 50 years) and ESS values showed significant differences:

In the ESS ≥ 16 group, 64.9% of patients were aged < 50 years, while in the ESS < 16 population almost half of all patients (47.80%) were over 50 years old (*p*<0.001) (Table 1).

**Table 1:** Demographic data of the study population. ESS: extremity severity score, GCS: Glasgow coma Scale, AIS: abbreviated injury Scale, NISS: new injury severity score, RISC2: revised injury severity score 2. N total: cases with available data. SD: standard deviation

	*N* total	ESS < 16	ESS ≥ 16	*p*-Value
Total [n]	1227	*n* = 822	*n* = 405	
Gender male [%/n]	1227	74,3%/*n* = 611	70,8%/*n* = 287	0,219
Age [Mean/SD]	1227	48.31/±21.82	42,16/±19,05	**< 0**,**001**
Age < 50 years [%/n]	1227	52,20%/*n* = 429	64,90%/*n* = 263	**< 0**,**001**
GCS [Mean/SD]	1203	10,03/±4,90	10,83/±4,75	**0**,**046**
GCS age ≤ 50 [Mean/SD]	679	9,88/±4,95	10,54/±4,76	0,089
GCS age > 50 [Mean/SD]	524	10,19/±4,84	11,37/±4,69	**0**,**013**
AIS head [Mean/SD]	1043	2.90/± 1.99	1.76/± 1.87	0,400
AIS thorax [Mean/SD]	1043	2,18/±1,80	2,53/±1,65	**< 0**,**001**
AIS abdomen [Mean/SD]	1043	0,73/±1,34	1,45/±1,60	**< 0**,**001**
ISS [Mean/SD]	1227	30,34/±15,27	34,24/±15,4	**0**,**038**
NISS [Mean/SD]	1227	38,20/±17,35	37,71/±15,88	**0**,**026**
RISC2 prognosis of death [Mean/SD]	1227	23,26/±32,56	21,48/±32,02	0,364

Regarding the injury pattern, the ESS ≥ 16 group showed significantly higher values for ISS, AIS thorax and AIS abdomen. In contrast, the NISS showed a significant difference of 0.49 with a higher value for the ESS < 16 group. The expected lethality, represented by the RISC2 showed a higher value for the ESS < 16 group, however this did not reach statistical significance. When evaluating the GCS, patients who suffered a severe limb injury showed an average score 0.8 points higher (ESS ≥ 16 10.83 vs. ESS < 16 10.03; *p* = 0,046). In the analysis of the subgroups divided by age, the younger patients (age ≤ 50 years) showed no significant differences (ESS ≥ 16 10.54 vs. ESS < 16 9.88; *p* = 0.089), while patients aged > 50 years had a significantly higher GCS value for severe limb injury (ESS ≥ 16 11.37 vs. ESS < 16 10.19, *p* = 0.013).

Regarding the cause of injury, a higher percentage of patients with an ESS ≥ 16 were involved in accidents with cars/trucks (40.49%, *n* = 164 vs. 31.17%, *n* = 255; *p* = 0.001) and motorcycles (24.94%, *n* = 101 vs. 12.71%, *n* = 104; *p* < 0.001) than in the comparison group. Only in the subgroup with an age ≤ 50 and accidents involving cars/trucks was there no significant difference. Particularly striking in this subgroup analysis is the ESS < 16 group with an age ≤ 50 years, in which cyclists were significantly more frequently involved in accidents (ESS ≥ 16: 1.90%, *n* = 5 vs. ESS < 16: 8.20%, *n* = 35; *p* < 0.001). Patients with less severely injured extremities were found with 20.56% (*n* = 168) in the group of falls with a fall height of less than three meters (ESS ≥ 16: 1.48%, *n* = 6, *p* < 0.001). But as expected, we also see significantly more low falls in the 50 + age group.

There were also differences in pre-hospital care and patient care in the trauma bay. For example, significantly (*p* = 0.001) more time (ESS ≥ 16: 63.37 min/ESS < 16: 57.41 min) was required for patient care and transportation to the appropriate destination hospital in the case of severe limb injury than in the comparison population. The need for infusion therapy (*p* < 0.001) and catecholamine administration (*p* = 0.005) was also significantly increased in the ESS ≥ 16 group, while the time to CT imaging (*p* < 0.001) was shorter in patients with less severely injured extremities (ESS < 16) (Table 2*).*

**Table 2:** Prehospital times and parameters as well as care in the trauma room in comparison of the two study populations ESS ≥ 16 and ESS < 16. HEMS: helicopter emergency medical service, eFAST: extended focused assessment with sonography for Trauma, CT: computer Tomography, ESS: extremity severity Score. N total: cases with available data. SD: standard deviation

	*N *total	ESS < 16	ESS ≥ 16	*p*-Value
		*n* = 822	*n* = 405	
Minutes from accident to ER [Mean/SD]	819	80,53/±29,24	85,67/±28,87	**0**,**016**
Minutes from emergency physician on scene to ER [Mean/SD]	743	57,41/±23,31	63,37/±24,92	**0**,**001**
HEMS transportation [%/n]	1216	61,3%/*n* = 498	72,2%/*n* = 291	**0**,**004**
Intubation preclinical [%/n]	1082	53,96%/*n* = 388	59,23%/*n* = 215	0,208
Infusion crystalloid preclinical [Mean/SD]	1055	828,04/± 757,36	1083,96/± 699,53	**< 0**,**001**
Infusion therapy colloid preclinical [Mean/SD]	1053	190,09/± 369,32	389,21/± 469,11	**< 0**,**001**
Catecholamines preclinical [Mean/SD]	1062	18,00%/*n* = 148	25,19%/*n* = 102	**0**,**005**
Minutes until eFAST trauma bay [Mean/SD]	857	6,83/±6,68	6,51/±2,63	0,430
Minutes to CT [Mean/SD]	928	16,84/±7,61	19,34/± 8,16	**< 0**,**001**
Minutes total trauma bay [Mean/SD]	1021	65,91/±34,81	68,41/±36,40	0,287
Infusion crystalloid trauma bay [Mean/SD]	1060	617,78/± 567,10	692,97/± 618,48	**0**,**049**
Infusion therapy colloid trauma bay [Mean/SD]	1060	146,13/± 351,49	375,19/± 567,09	**< 0**,**001**
Catecholamines trauma bay [%/n]	1065	35,65%/*n* = 293	49,63%/*n* = 201	**< 0**,**001**

## Clinical Course/Secondary endpoints

Looking at the transfusion frequency in the first 24 h, the subgroups age > 50 years showed a highly significant difference (ESS ≥ 16: 59.86%/ESS < 16: 20.37%, *p* ≤ 0.001) (Table 3). In addition, patients with severe limb injury were significantly (*p* ≤ 0.001) longer (12.10 days) in the intensive care unit than patients in the comparison group ESS < 16 (8.91 days). Looking at the time that patients required mechanical ventilation, the mean value was also higher in the ESS ≥ 16 group (6.05 days) compared to the comparison group (5.34 days), although this difference was not statistically significant (*p* = 0.814). A highly significant (*p* ≤ 0.001) difference was found when looking at the total length of stay in hospital. Patients with severe extremity injury required an average length of stay of 24.56 days, which was about 8.07 days longer than the values for the ESS < 16 group (16.49 days) (Table 3). In a comparison of the age subgroups, the length of stay in the intensive care unit decreased with decreasing age but still showed a significant difference. Especially in the older age group (> 50 years), the total length of stay in hospital showed its largest range with a significant (*p* ≤ 0.001) difference of 8.80 days (ESS ≥ 16 vs. ESS < 16). It should be noted that patients with an age ≤ 50 years and an ESS < 16 died significantly (*p* = 0.001) earlier than patients in the comparison group ESS ≥ 16. This can be explained by an increased incidence of most severe brain injuries (AIS ≥ 5) in this subgroup (ESS < 16: 90/24.06%; ESS ≥ 16: 31/13.47% [%/n]). When looking at the surgical treatments, it was shown that the ESS ≥ 16 group and its subgroups required significantly more operations (Table 3).

**Table 3:** Parameters of risk and influencing factors of severe limb injury, clinical course, secondary endpoints. PRBC: packed red blood cell concentrates, ICU: intensive care unit, ESS: extremity severity Score. N total: cases with available data. SD: standard deviation*.*

	Age	*N* total	ESS < 16	ESS ≥ 16	*p*-Value
PRBC administration first 24 h [%/n]	**total**	1227	20,31%/*n* = 167	50,62%/*n* = 205	**≤ 0**,**001**
	**≤ 50 years**	692	20,28%/*n* = 87	45,63%/*n* = 120	**≤ 0**,**001**
	**> 50 years**	535	20,37%/*n* = 80	59,86%/*n* = 85	**≤ 0**,**001**
Number of PRBC ≥ 10 first 24 h [Mean/SD]	**total**	61	15,57/±6,94	25,09/±15,70	**≤ 0**,**001**
	**≤ 50 years**	39	15,25/±6,54	27,24/±17,34	**0**,**032**
	**> 50 years**	22	16,00/±8,05	20,91/±11,23	0,342
Ventilation duration (days) [Mean/SD]	**total**	1188	5,34/±7,91	6,05/±8,24	0,814
	**≤ 50 years**	671	5,13/±7,17	5,63/±7,73	0,390
	**> 50 years**	517	5,56/±8,64	6,86/±9,11	0,142
ICU length of stay (days) [Mean/SD]	**total**	1223	8,91/±10,58	12,10/±14,556	**≤ 0**,**001**
	**≤ 50 years**	690	8,75/±10,47	11,61/±14,42	**0**,**003**
	**> 50 years**	533	9,09/±10,58	13,00/±14,81	**≤ 0**,**001**
Total length of stay in hospital (days) [Mean/SD]	**total**	1129	16,49/±14,68	24,56/±18,716	**≤ 0**,**001**
	**≤ 50 years**	604	16,84/±14,42	24,40/±18,33	**≤ 0**,**001**
	**> 50 years**	439	16,08/±14,99	24,88/±19,54	**≤ 0**,**001**
Operations per patient [Mean/SD]	**total**	1227	2,47/±3,06	7,64/±7,81	**≤ 0**,**001**
	**≤ 50 years**	692	2,90/±3,40	7,94/±8,48	**≤ 0**,**001**
	**> 50 years**	535	2,01/±2,55	7,10/±6,37	**≤ 0**,**001**
Time to death in days [Mean/SD]	**total**	258	4,24/±6,82	3,43/±6,24	0,188
	**≤ 50 years**	103	1,64/±2,78	3,11/±4,51	**0**,**022**
	**> 50 years**	155	5,61/±7,85	8,81/±7,91	0,123

## Primary endpoint: Lethality

With regard to lethality, the percentages of deaths did not show a significant difference neither for the two injury severity subgroups nor for the age subgroups. The ESS ≥ 16 subgroup of ≤ 50-year-olds showed a slightly increased mortality compared to ESS < 16, while overall and in the over 50 s the ESS < 16 subgroup showed an increased mortality. Similarly, the SMR, calculated with the RISC2, showed comparable results, again with a slightly better outcome for the ESS ≥ 16 groups (Table 4). The hypothesis that multiple limb injury causes higher mortality had to be rejected (Table 4).

**Table 4:** Primary endpoint lethality and SMR - Standardized mortality Ratio, ESS: extremity severity score

		*N* total	Deceased [*n*]	Deceased [%]	Value	*p*-Value
Lethality	**ESS < 16**	822	185	22,50%		0,070
	**ESS ≥ 16**	405	73	18,00%		
Lethality Age ≤ 50	**ESS < 16**	429	63	14,70%		0,851
	**ESS ≥ 16**	263	40	15,21%		
Lethality Age > 50	**ESS < 16**	393	122	31,04%		0,079
	**ESS ≥ 16**	142	33	23,24%		
SMR	**ESS < 16**	637			0,967	0,235
	**ESS ≥ 16**	332			0,838	
SMR Age ≤ 50	**ESS < 16**	429			0,836	0,197
	**ESS ≥ 16**	263			0,785	
SMR Age > 50	**ESS < 16**	393			1,040	0,376
	**ESS ≥ 16**	142			0,906	

Significant differences were found when examining the causes of death (Table 5). In both ESS groups, traumatic brain injury was the most common cause of death (ESS < 16: *n* = 97/52.43%; ESS ≥ 16: *n* = 27/36.99%). This was followed in the ESS ≥ 16 group by exsanguination, which led to death in 15.07% of patients (*n* = 11). In the comparison group, this was the cause of death in 7.03% (*n* = 13) of patients (*p* = 0.031). Multi-organ failure ranked third in frequency in both groups. While 13.70% of all patients (*n* = 10) in the ESS ≥ 16 population died from this cause, this cause was responsible for mortality in 4.86% (*n* = 9) of cases in the ESS < 16 population, a difference that can be interpreted as a trend (Table 5*)*. The analysis of the two subgroups according to age (≤ 50/> 50 years) showed that patients with an older age and ESS ≥ 16 died significantly (*p* = 0.007) more frequently from exsanguination than the comparison group. However, more than 54% of the older ESS < 16 group died of a TBI, significantly more than in the comparison group (24,24%).

**Table 5:** Causes of death. TBI: traumatic brain injury; SIRS: systemic inflammatory response Syndrome

	Age	ESS < 16	ESS ≥ 16	*p*-Value
		*n* = 185 *n*_age≤50_= 63 *n*_age>50_= 122	*n* = 73 *n*_age≤50_= 40 *n*_age>50_= 33	
Cause of death TBI [%/n]	**total**	52,43%/*n* = 97	36,99%/*n* = 27	0,004
	**≤ 50 years**	47,62%/*n* = 30	47,50%/*n* = 19	0,590
	**> 50 years**	54,92%/*n* = 67	24,24%/*n* = 8	**0**,**002**
Cause of death exsanguination [%/n]	**total**	7,03%/*n* = 13	15,07%/*n* = 11	**0**,**031**
	**≤ 50 years**	14,29%/*n* = 9	12,5%/*n* = 5	0,613
	**> 50 years**	3,28%/*n* = 4	18,18%/*n* = 6	**0**,**007**
Cause of death Multi-organ failure [%/n]	**total**	4,86%/*n* = 9	13,70%/*n* = 10	0,055
	**≤ 50 years**	1,59%/*n* = 1	10,0%/*n* = 4	0,084
	**> 50 years**	6,57%/*n* = 8	18,18%/*n* = 6	0,092
Cause of death SIRS/sepsis [%/n]	**total**	0,54%/*n* = 1	2,74%/*n* = 2	0,449
	**≤ 50 years**	0,00%/*n* = 0	2,50%/*n* = 1	0,241
	**> 50 years**	1,64%/*n* = 2	3,03%/*n* = 1	0,705
Cause of death other [%/n]	**total**	6,49%/*n* = 12	9,59%/*n* = 7	0,670
	**≤ 50 years**	9,52%/*n* = 6	5,00%/*n* = 2	0,317
	**> 50 years**	4,92%/*n* = 6	15,15%/*n* = 5	0,091

## Logistic regression

The logistic regression model was set up to predict mortality. It was statistically significant, χ² [6] = 345.392, *p* < 0.001, with a good variance explanation of Nagelkerke’s *R²* = 0.458, according to the recommendations of Backhaus et al. [26]. The overall percentage of correct classification was 85.40%, with a sensitivity of 93.50% and a specificity of 52.40%. Of the six predictor variables included in the logistic regression model, five were significant. Age at the time of accident in years, GCS score, AIS head, pre-hospital shock defined as RR ≤ 90mmHg and administration of more than 10 PRBCs had a significant impact (*p* < 0.001) on the predictive performance of the model. A younger age at the time of the accident had a protective effect on survival. The increase in age by one year had an odds ratio of 1.044 (95% CI [1.033, 1.054]). For the scoring values, the GCS showed a risk of death at higher values with an odds ratio of 0.818 (95% CI [0.776, 0.863]), which means a high risk at lower values. The inverse odds for a decrease in GCS is 1,222. The AIS for the head region shows an increased risk of death the higher the injury is classified (95% CI [1.244, 1.629]). Both increased PRBC administration with more than 10 PRBCs in the first 24 h (odds of 5.561 (95% CI [2.313, 13.370])) and pre-hospital shock, defined as systolic blood pressure ≤ 90mmHg (odds 3.724 (95% CI [2.255, 6.150])) are considered a risk for death. The ESS ≥ 16 showed no significant results. All model coefficients and odds ratios can be found in Table 6.

**Table 6:** Reults of the logistic binomial regression. GCS: Glasgow coma Scale; AIS: abbreviated injury Score; PRBC: packed red blood Cells, ESS: extremity severity Score. CI: confidence interval

	B	SD	Wald	*p*-Value	Odds Ratio	95% CI for odds ratio	
						Lower	Upper
Age at the time of the accident [years]	−0.043	0.005	67.794	**< 0.001**	1.044	1.033	1.054
GCS	−0.200	0.027	54.782	**< 0.001**	0.818	0.776	0.863
AIS head	0.353	0.069	26.404	**< 0.001**	1.424	1.244	1.629
Blood pressure syst. ≤90mmHg	1.315	0.256	26.380	**< 0.001**	3.724	2.255	6.150
PRBC ≥ 10	1.716	0.448	14.692	**< 0.001**	5.561	2.313	13.370
ESS	−0,009	0.008	1.236	0.266	0.991	0.976	1.007
Constant	−3,257	0.489	44.390	< 0,001	0.039		

## Discussion

To the best of our knowledge, this is the first study to approach the effects of multiple extremity injury using a dedicated, separate new scoring system. The key finding is the confirmation of the assumption that multiple limb injuries tend to have no effect on the primary endpoint of mortality.

This underlines the recommendations in the S3 guideline on polytrauma/severe injury care and the ATLS manual [4, 5]. Ernstberger et al. showed that the mortality rate for patients with a severe injury to the extremities (18.00%) in a Level I center was lower than the mortality rate for all trauma room patients (23.00%) [27]. If one compares the standardized mortality rate of this study with the results of the 10-year comparison of the TraumaRegister DGU^®^ (SMR >1), a possible survival advantage for patients of the study population ESS ≥ 16 (SMR: 0.838) and especially patients with an age ≤ 50 years (SMR: 0.785) could be derived [21]. Reasons for this could be related to the type of the accident, as young patients with severe limb injuries are more likely to be involved in traffic accidents, while older patients with an ESS < 16 most commonly suffer a fall from a low height.

Nevertheless, severe bleeding in the event of serious injuries to the extremities must not be ignored. Severe, uncontrollable bleeding following serious trauma remains one of the main causes of death in traumatology [28–31]. Severe bleeding leads to hemorrhagic shock situations, which are associated with a high mortality rate. Bleeding in the abdomen and thorax is often cited as the reason for this. The increased focus of the guidelines [4, 5] on extremity injuries with heavy blood loss is supported by the results of our study. Particularly in the ESS population ≥ 16, exsanguination is a cause of death that can also be caused by injuries to the extremities, which underlines the recommendation to control bleeding. It should be noted that injuries to the thorax and abdomen, measured via the AIS, are also significantly higher in the ESS ≥ 16 group. These are to be regarded as predictors of a severe trunk injury. Due to the severe injury to the extremities, the total blood loss now adds up and is significantly higher than if this were to occur only in the area of the thorax and abdomen injuries, which are among the known relevant bleeding areas [4, 32]. A severe injury to the extremities is often related to severe injuries in the thorax and abdomen regions and these patients have a high risk of bleeding to death.

These figures are also supported by the significantly increased need for blood transfusions in patients with severe limb injuries. It should be emphasized here that transfusions are required to stabilize patients, particularly in the older age group, although no significant difference in injury severity can be demonstrated in this subgroup using the ISS. The evaluation by El Mestoui et al. should be mentioned here, which showed similar figures at a Dutch level I trauma center, with severe bleeding being the second most common cause of death [33]. However, it should be borne in mind that the incidence of multi-organ failure increases with each administration of an erythrocyte concentrate in polytrauma patients, as Patel et al. found in a 2014 review including 40 empirical studies [34]. The incidence of multi-organ failure (MOF) is significantly higher than the actual mortality rate. Of the 30,000 patients examined in the study by Fröhlich et al. 32.70% developed an MOF. In addition to the patient’s age, particular importance should also be attached to the GCS and mass transfusion in these patients [35].

A clearly significant (*p* ≤ 0.001) difference was seen when looking more closely at the patient’s total length of stay in hospital. In particular, the oftentimes complex therapy and the need for multiple surgical procedures appear to be predictors of a longer hospital stay. The longer time spent in the intensive care unit for patients in the ESS ≥ 16 group also played a role in these results. It should be noted that a significant difference in time to death was only observed in the subpopulation of young patients. When looking at the study as a whole, no direct effect can be derived from this variable, but it is an indication of the mortality rate of the other AIS regions. The increased need for intensive care and surgical interventions combined with the need for transfusions ultimately leads to prolonged clinical therapy.

The data from this study also showed a significant prolongation of monitoring in the intensive care units in the group of patients with ESS ≥ 16. A comparison of these times with the 10-year average of the TraumaRegister DGU^®^ shows that patients with ESS ≥ 16 require almost twice as long an intensive care stay as the average for all multiple trauma patients [21]. This duration is certainly not only due to physiological dysfunctions caused by the fractures, but also to the significantly increased need for surgical measures, which prolong the intensive care period.

Although not the direct focus of this study, minor trauma in older people must nevertheless be mentioned. As Pape et al. were able to demonstrate [36], the focus in older patients should be on traumatic brain injury. In our study, traumatic brain injury was the main cause of death in the ESS < 16 study group, which was significantly older than the patients in the ESS ≥ 16 group in the overall study population. This was even more pronounced in the ESS < 16 and age >50 subgroup.

A low fall has a significantly higher risk in old age; craniocerebral trauma in particular must be taken into account here [36]. While the entire patient population of this study is comparable to all patients who suffer a multiple trauma in Germany, multiple limb injuries are considered to be a rather low risk in older patients. The proximal femur fracture has an ESS value of 9. However, it should be emphasized that the values of the ESS ≥ 16 group showed a lower SMR across all age groups and in the study as a whole than the comparison group ESS < 16.

The regression model was unable to unmask any influence of multiple limb injury on mortality. Based on the results of the univariate analyses, as expected, a younger age at the time of the accident has a protective effect on survival. The significant results for the GCS show the importance of the scoring systems. The preclinical shock and the need for transfusion also emphasize the importance of bleeding in polytraumatized patients and underline the lethality reason exsanguination again. Particularly in older patients, special attention should therefore be paid not only to the regions already considered important in the current literature, but also to severe bleeding as a risk factor. These results underline the calculations of the univariate analyses.

It can therefore be postulated that in severely injured patients with an ISS ≥ 16 according to the inclusion criteria of this study, the serious injury to the extremities has no direct influence on lethality, but the secondary endpoints associated with it, such as severe bleeding and complications, should not be disregarded.

## Limitations

This study has several limitations:

1. Single-Center Study
  The study was conducted at a well-trained Level I Trauma Center in Germany. We assume that the results can also be transferred to other Level I Trauma Centers internationally with predominantly blunt trauma as the accident mechanism. Centers with a significantly higher proportion of gunshot injuries will come to different results for exsanguination in the ESS < 16 range.
2. Retrospective study
  The retrospective design is inherent to registry studies. This study benefits from the fact that the survey team was present in every trauma room, the data was collected prospectively in the trauma room and all data records were checked using the dual control principle. Accordingly, this study has a very high quality for completeness and accuracy. Furthermore, the study is to be understood in the sense of health services research and represents the actual patient population of the study clinic over the period.
3. Non-recording of preclinical deaths
  Patients who died preclinically could not be included in the study. In terms of exsanguination and TBI, there could be shifts if the preclinically deceased could be included. However, this does not affect the clinical treatment. With a preclinical time of 60 min, a bias due to an excessively long rescue time from rough terrain or similar is also not to be expected.
4. Lack of comparative studies
  There are no studies in the current literature that dealt with multiple extremity injuries in combination with a scoring system such as the ESS in polytraumatized patients, so that a direct comparison with other studies was not possible.
5. Calculation of the ESS
  The ESS was calculated in analogy to the NISS. The three most severe bony injuries following AIS of the extremities and pelvis were squared and added together. Accordingly, the ESS has the same limitations as the NISS and the AIS. There may be better scoring systems for extremity fractures with mortality as the endpoint in the future.

Accordingly, further studies on multiple limb fractures should be conducted in different countries to confirm or refute our findings.

## Conclusion

Using a new scoring system for recording multiple extremity injuries, this study from a Level I hospital showed that multiple, serious extremity injuries have no influence on the lethality of severely injured patients and confirms ATLS and the German S3 guideline. Nevertheless, patients with multiple extremity fractures have significantly higher resource consumption and are high-risk patients. Future studies may investigate whether the results of this study can be transferred to other countries or hospitals with fewer resources.

## Data Availability

The dataset generated and analyzed during the current study is not publicly accessible but is available from the corresponding author on reasonable request.
